# Thrifty metabolic programming in rats is induced by both maternal undernutrition and postnatal leptin treatment, but masked in the presence of both: implications for models of developmental programming

**DOI:** 10.1186/1471-2164-15-49

**Published:** 2014-01-21

**Authors:** Peter JI Ellis, Tiffany J Morris, Benjamin M Skinner, Carole A Sargent, Mark H Vickers, Peter D Gluckman, Stewart Gilmour, Nabeel A Affara

**Affiliations:** 1 University of Cambridge Department of Pathology, Tennis Court Road, Cambridge CB2 1QP, UK; 2 UCL Cancer Institute, University College London, London WC1E 6BT, UK; 3 Liggins Institute and Gravida: National Centre for Growth and Development, University of Auckland, Auckland, New Zealand

**Keywords:** Leptin, Fetal programming, Development, Obesity, Thrifty phenotype, Antigen presentation, Inflammation

## Abstract

**Background:**

Maternal undernutrition leads to an increased risk of metabolic disorders in offspring including obesity and insulin resistance, thought to be due to a programmed thrifty phenotype which is inappropriate for a subsequent richer nutritional environment. In a rat model, both male and female offspring of undernourished mothers are programmed to become obese, however postnatal leptin treatment gives discordant results between males and females. Leptin treatment is able to rescue the adverse programming effects in the female offspring of undernourished mothers, but not in their male offspring. Additionally, in these rats, postnatal leptin treatment of offspring from normally-nourished mothers programmes their male offspring to develop obesity in later life, while there is no comparable effect in their female offspring.

**Results:**

We show by microarray analysis of the female liver transcriptome that both maternal undernutrition and postnatal leptin treatment independently induce a similar thrifty transcriptional programme affecting carbohydrate metabolism, amino acid metabolism and oxidative stress genes. Paradoxically, however, the combination of both stimuli restores a more normal transcriptional environment. This demonstrates that “leptin reversal” is a global phenomenon affecting all genes involved in fetal programming by maternal undernourishment and leptin treatment. The thrifty transcriptional programme was associated with pro-inflammatory markers and downregulation of adaptive immune mediators, particularly MHC class I genes, suggesting a deficit in antigen presentation in these offspring.

**Conclusions:**

We propose a revised model of developmental programming reconciling the male and female observations, in which there are two competing programmes which collectively drive liver transcription. The first element is a thrifty metabolic phenotype induced by early life growth restriction independently of leptin levels. The second is a homeostatic set point calibrated in response to postnatal leptin surge, which is able to over-ride the metabolic programme. This “calibration model” for the postnatal leptin surge, if applicable in humans, may have implications for understanding responses to catch-up growth in infants. Additionally, the identification of an antigen presentation deficit associated with metabolic thriftiness may relate to a previously observed correlation between birth season (a proxy for gestational undernutrition) and infectious disease mortality in rural African communities.

## Background

Alterations in nutrition during fetal and perinatal life are linked to adverse health outcomes in offspring in adulthood, this being known as the Developmental Origins of Health and Disease (DOHaD) paradigm [1]. In particular, data from epidemiological cohorts and a range of animal models has shown that maternal undernutrition results in an increased risk of obesity and metabolic disease in offspring in later life [2,3]. The most commonly used model of maternal undernutrition is in the rat where studies have utilised moderate through to severe undernutrition (20-70% calorie restricted diets) to examine mechanisms underlying the programming of later disease risk [4-6]. Protein undernutrition appears to be particularly important, with low protein isocaloric diet models showing similar effects to caloric restriction models in both rat and mouse [7,8].

The adverse phenotypic outcomes, particularly those related to metabolic abnormalities, are thought to result in part from the mismatch between a deprived early nutritional environment which programmes a “thrifty phenotype” (also called a “predictive adaptive response”), and a richer later environment for which this metabolic programme is inappropriate [9-11]. In addition to the metabolic sequelae of maternal undernutrition, in some circumstances such deprivation can have long-lasting consequences for the immune system, for example the greatly increased prevalence of infectious disease among Gambian individuals born in the “hungry season” [12-14]. This latter result was however not replicated in studies in rural Bangladesh or Senegal [15,16], indicating a wide degree of heterogeneity in the immune response to maternal undernutrition.

We have previously shown in a rat model (70% caloric restriction) that neonatal administration of the adipokine leptin reverses the metabolic abnormalities seen in the offspring of undernourished mothers [17,18]. Both male and female offspring of mothers subject to maternal undernutrition during pregnancy developed increased adiposity and markers of the metabolic syndrome, particularly when fed a high fat diet postweaning. Neonatal treatment with leptin during a key period of developmental plasticity (postnatal age 3-13 days) reversed the programmed phenotype and restored near normal metabolic parameters [17,18]. This observation was associated with a reversal of the direction of the leptin response (hereafter “leptin reversal”) in the offspring of undernourished mothers (‘UN offspring’). Neonatal leptin treatment increased adult levels of 11β-HSD2 transcription in the livers of normally-nourished pups, but decreased it in livers of the UN offspring: similar leptin reversal effects on both transcription and promoter methylation were observed for PPARα, GR and PEPCK [19].

In this follow-up analysis, we carried out whole-genome expression profiling of liver RNA from the female experimental series previously described, in order to fully characterise the global hepatic responses to maternal undernutrition (AD/UN = ad-libitum fed or undernourished mothers), postnatal leptin administration (Lep/Sal = leptin or saline control given postnatally), level of postweaning diet (Chow/HF = normal or high fat diet), and the interactions between these three factors. In particular, we aimed to determine:

1. The extent of the leptin reversal–whether it affected all leptin-regulated genes, or a specific subset of these.

2. Which of the genes affected by the leptin reversal were associated with the metabolic syndrome seen in UN/Sal/HF offspring.

3. Whether there were precursor changes seen in UN/Sal/Chow offspring, which could potentially be markers for those at risk of developing metabolic syndrome and/or diabetes.

4. Whether there were other non-metabolic genes and pathways affected by maternal undernutrition and/or leptin reversal which might therefore be implicated in further phenotypes, We were particularly interested in pathways relating to immune system regulation, given the programmed effects (in human) of undernutrition on infectious disease prevalence.

The transcriptional data indicated a significant similarity between the AD/Lep cohorts and the UN/Sal cohorts. This was unexpected since the phenotypic outcomes differ markedly between these two interventions, in a sex-specific manner. In particular, in females UN/Sal offspring become severely obese when fed a high fat diet, while AD/Lep do not: the reverse is the case in males. These transcriptional findings therefore led us to re-examine our previously published growth curves for both the male and female experimental series. Previously, we had analysed the preweaning and postweaning data separately. In this re-analysis we focus on the weaning period itself: a critical juncture during which growing pups take over full responsibility for their own nutritional intake. We find that growth trajectories differ between male and female pups during weaning, and interpret the male and female data collectively as the consequences of two competing programmes: a thriftiness programme which governs the efficiency of fuel, and a homeostatic set point governing body composition.

## Results

We used Illumina oligonucleotide arrays to perform expression profiling on RNA extracted from livers of female rats in 8 treatment groups (i.e. all combinations of ad-libitum-fed or undernourished mothers, postnatal leptin treatment or saline, postweaning high fat or normal chow diet), n = 8 animals per group. An initial one-way ANOVA analysis was used to select all genes significantly differentially expressed in at least one of the treatment groups. 2221/8497 (26.1%) of liver-expressed genes were called as significant in this analysis, with 1069 of these showing at least 1.25 fold change in transcript abundance between the highest-and lowest-expressing treatment groups. Three-factor ANOVA analysis was subsequently used to categorise the genes according to which individual experimental factors, or interactions between factors, were significant in each case (Table 1 and Additional file 1: Table S1).

**Table 1:** Numbers of significantly regulated genes, categorised according to which factors and/or interactions were significant in the three-way ANOVA

**Category**	**Significant genes**	**Biological interpretation**
**A** Maternal nutrition	368 (151)	Maternal nutritional status has an effect independent of leptin treatment and postweaning diet
**B** Leptin treatment	200 (90)	Leptin treatment has an effect independent of maternal nutritional status and postweaning diet
**C** Postweaning diet	990 (531)	Postweaning diet has an effect independent of maternal nutritional status and leptin treatment
**AB** (2-way interaction)	949 (503)	The effect of leptin treatment is dependent on maternal nutritional status (or vice versa)
**AC** (2-way interaction)	96 (18)	The effect of postweaning diet is dependent on maternal nutritional status (or vice versa)
**BC** (2-way interaction)	127 (74)	The effect of postweaning diet is dependent on leptin treatment (or vice versa)
**ABC** (3-way interaction)	7 (3)	The effects of all three factors are mutually dependent
**Uncategorised**	128 (59)	Although significant changes were detected by one-way ANOVA, no individual factor or interaction term was subsequently significant in a three-factor ANOVA

Bracketed figures for each category indicate the number of genes in the category that showed at least 1.25 fold change in transcript abundance across the data set as a whole. Additional file 2: Table S2 shows expression data for all genes falling into each category.

### Maternal undernutrition and postnatal leptin treatment predominantly affect the same target set

Whether considering all significant transcripts, or only those showing more than 1.25 fold change, the largest group of transcriptional changes (category C) represents the non-interacting effects of postweaning diet–unsurprising as diet at sacrifice directly affects the animals’ metabolic status and consequently hepatic expression profiles. However, category AB (significant regulation by the interaction between maternal nutrition and leptin treatment) contained very nearly the same number of transcripts, indicating that (a) these two factors are able to collectively programme transcriptional responses occurring many days post-intervention, and (b) these programming effects are equally as important as postweaning diet in determining the final liver expression profile. Importantly, category AB contains many more genes than either category A or B alone, indicating that maternal undernutrition and postnatal leptin treatment largely affect the same genes and that these effects are mutually dependent.

### Phenotypic data for the programmed cohorts also show significant interactions between maternal diet and postnatal leptin treatment

The phenotypic characterisation of this experimental series was expanded and re-examined, again using three-factor ANOVA (Additional file 2: Table S2). Importantly, while total body fat percentage is increased as expected by a postweaning high fat diet (postweaning diet F_1,56_ = 150.5, p = 1.67 × 10^-17^), it also shows a significant interaction between maternal undernutrition and postnatal leptin treatment (AB interaction F_1,56_ = 22.25, p = 1.63 × 10^-5^). Total body fat percentage is increased by postnatal leptin treatment (AD/Lep) and maternal undernutrition (UN/Sal) but decreased by the combination of both (UN/Lep). Plasma leptin levels follow the same pattern as body fat percent (AB interaction F_1,56_ = 11.32, p = 1.39 × 10^-3^), and fasting C-peptide levels also show a highly significant AB interaction term (AB interaction F_1,56_ = 13.13, p = 6.29 × 10^-4^) and a similar profile of dysregulation. The AB interaction term for fasting insulin levels was just significant before FDR correction but not significant after correction. Plasma ghrelin and other metabolic parameters (liver glycerol and triglycerides, plasma glycerol, triglycerides and free fatty acids) were all predominantly regulated by postweaning diet.

### There is a complex spectrum of “reversal” interactions between transcriptional programming by maternal nutritional status and by postnatal leptin treatment

The interactions between maternal nutrition status and leptin treatment could be either synergistic or opposing. For the 949 genes in category AB, we compared the expression change induced by leptin treatment in pups born to UN mothers (average Lep/Sal expression ratio averaged across UN cohorts) to that in pups of ad libitum fed mothers (the same ratio averaged across AD cohorts) to determine the interaction type. The large majority of genes (871/949 = 91.8%) showed a “leptin reversal” pattern where the average Lep/Sal ratio in UN cohorts was opposite in sign to that in AD cohorts. Only a small proportion of genes showed a synergistic interaction (57/949 = 6.0%) or a partially opposing interaction (21/949 = 2.2%), defined as genes where the average Lep/Sal ratio in UN cohorts was the same sign as in AD cohorts and of higher magnitude (synergistic) or lower magnitude (partially opposing).

**Figure 1:**
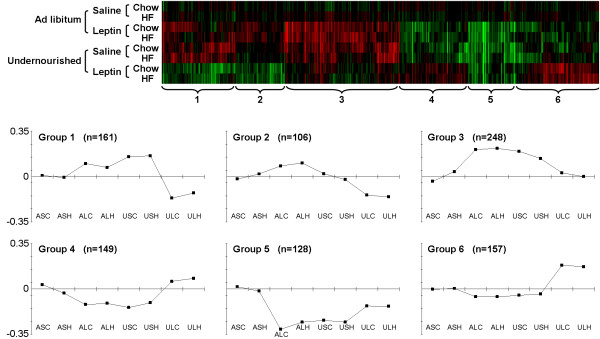
**Heatmap showing hierarchical clustering of the 949 genes with a significant interaction between maternal diet and leptin treatment, after subtracting out the effect of postweaning diet.** Line graphs show the mean (centroid) expression profile for each cluster.

Hierarchical clustering of the category AB genes was performed to determine whether there was evidence for a binary polyphenism as suggested by Gluckman et al. [19]. Figure 1 is a heatmap of these 949 genes showing the residual expression changes attributable to the combination of maternal diet and/or leptin treatment. Interacting genes fell into six broad groups of response pattern, all of which show “leptin reversal” since the direction of leptin-related change is opposite in AD and UN cohorts, but with important differences in the nature of the reversal.

Group 1 and 3 genes are induced both by maternal undernutrition and by leptin treatment (compare USC/H and ALC/H to ASC/H), however the combination of both factors (compare ULC/H to ASC/H) causes either no induction of these genes (Group 3) or even a net repression (Group 1). Group 2 genes show no net effect of maternal undernutrition on its own (compare USC/H to ASC/H), but are induced by leptin in AD cohorts (compare ALC/H to ASC/H) and repressed by leptin in UN cohorts (compare ULC/H to USC/H). Group 4 and 5 genes are repressed by both factors acting alone, but show a reduced repression or even a net induction when both factors are present (opposite to groups 1 and 3). Group 6 genes are induced specifically by the combination of both factors.

The vast majority of AB-interacting genes show similar changes in AD/Lep cohorts and in UN/Sal cohorts relative to the control AD/Sal cohorts. These results therefore support the hypothesis of a binary polyphenism in liver transcriptional activity, where the phenotypic switch is seen in response both to undernutrition and to postnatal leptin treatment, but not the combination of both.

### Functional annotation analysis of leptin reversal genes

DAVID [20] was used to collate functional annotations into annotation groups associated with each of the identified AB-interacting gene clusters by combining data from multiple databases including Gene Ontology, Swiss-Prot, Uni-Prot, Protein Information Resource, InterPro and KEGG pathways (Table 2 and Additional file 3: Table S3). Each annotation group has an enrichment score denoting whether it is significantly associated with any given gene cluster (enrichment score of ≥ 2 represents a p-value ≤ 0.01 after correction for multiple testing). This analysis revealed several functional shifts associated with maternal undernutrition and leptin treatment.

**Table 2:** Functional clusters over-represented among genes with a significant interaction between maternal undernutrition and postnatal leptin treatment

**Expression pattern**	**Annotation group**	**Genes with AB interaction**
Induced by maternal undernutrition and by leptin treatment, but not by both combined (Groups 1 & 3)	Mitochondrially-targeted genes (enrichment score 2.64 → 4.55)	*Aadac*; *Aadat*; *Abcb11*; *Acat2*; *Acsl3*; *Acsl5*; *Adhfe1*; *Agtr1a*; *Aifm1*; *Ak3l1*; *Aldh1b1*; *Aldh1l2*; *Atad1*; *Atp6v1a1*; *Bche*; *Bckdhb*; *Bphl*; *Cabc1*; *Clpx*; *Crls1*; *Cry1*; *Cyb5r3*; *Cyp2r1*; *Dhcr24*; *Dhtkd1*; *Dnaja3*; *Fam82a*; *Fut8*; *Glud1*; *Gm2a*; *Golph3*; *Gpam*; *Gpd2*; *Hmbs*; *Hmgcr*; *NIT2*; *LOC501346*; *LOC685778*; *LOC688587*; *TTC19*; *Lrp5*; *Lypla1*; *Maoa*; *Maob*; *Me1*; *Mgst1*; *Mpv17l*; *Mrpl18*; *Mrpl50*; *Mterfd3*; *Mtfr1*; *Nnt*; *Nsdhl*; *Otc*; *Pck2*; *Pdhb*; *Qdpr*; *Rab5a*; *RGD1308114*; *RGD1309676*; *PEX1*; *MYO6*; *MUT*; *TMEM70*; *Rilp*; *Rpl10a*; *Sars2*; *Scp2*; *Scrn1*; *Sec22b*; *Senp2*; *Sfxn5*; *Slc16a1*; *Slc25a17*; *Slc35a3*; *Slc35b3*; *Sod2*; *St3gal6*; *Synj2bp*; *Tbc1d15*; *Tm7sf2*; *Tmpo*; *Tnks2*; *Txndc1*; *Uros*
	Microsome/peroxisome/cytochrome p450 pathway (enrichment score 2.03 → 4.46)	*A1cf*; *Aadac*; *Abca6*; *Abcb11*; *Acsl3*; *Acsl5*; *Agl*; *Aifm1*; *Aldh1a1*; *Baat*; *Bche*; *Cabc1*; *Cadm1*; *Clcn2*; *Cnbp*; *Csnk1g3*; *Ctsc*; *Cyb5r3*; *Cyp2d22*; *Cyp2d5*; *Cyp2r1*; *Dhcr24*; *Dio1*; *Dpys*; *Ephx1*; *Erap1*; *Fmr1*; *Gbe1*; *Hmgcr*; *Isoc1*; *Maoa*; *Mbl1*; *Mbl2*; *Me1*; *Mgst1*; *Mpv17l*; *Nsdhl*; *Nt5e*; *Pck2*; *Pgd*; *Pgk1*; *Pipox*; *Ppp1r3b*; *Ptprf*; *Pygl*; *Rab5a*; *Rdh3*; *PEX1*; *GNPNAT1*; *Rgs16*; *Sat1*; *Scp2*; *Sec22b*; *Slc10a1*; *Slc25a17*; *Sod2*; *Sord*; *Srd5a1*; *Tm7sf2*; *Tmco1*; *Tmed5*; *Tnks2*; *Txndc1*; *Ugt2b*; *Ugt2b17*; *Ugt2b36*
	Steroid synthesis and response (enrichment score 1.86 → 2.69)	*Aadac*; *Abcb11*; *Abhd5*; *Acsl3*; *Acsl5*; *Afp*; *Agl*; *Agtr1a*; *Ak3l1*; *Aldh1a1*; *Angptl4*; *Avpr1a*; *Baat*; *Bche*; *Bckdhb*; *Btg1*; *Cfb*; *Crls1*; *Ctsc*; *Cyb5r3*; *Cyp2d22*; *Cyp2r1*; *Dhcr24*; *Dnaja3*; *Dnajb5*; *Ephx1*; *Erap1*; *Foxo1a*; *Fut8*; *Gpam*; *Hmgcr*; *Ihh*; *Laptm4b*; *LOC501346*; *ANGPTL3*; *LOC686548*; *LOC688587*; *Lrp5*; *Me1*; *Mgst1*; *Mif*; *Nfe2l2*; *Nsdhl*; *Osbp*; *Pck2*; *Pggt1b*; *Prkaa1*; *Rab5a*; *Rcan1*; *RGD1560513*; *MYO6*; *STARD5*; *Scrn1*; *Sec22b*; *Senp2*; *Serpina3m*; *Serpinf1*; *Slc34a2*; *Slc35a3*; *Smad2*; *Sord*; *Srd5a1*; *St3gal6*; *Sult1a1*; *Tm7sf2*; *Tmpo*; *Tnks2*; *Txndc1*; *Uros*
	Starch metabolism (enrichment score 3.09)	*Adh6*; *Agl*; *Aldh1a1*; *Aldh1b1*; *Amy1a*; *Cyp2d22*; *Cyp2d5*; *Dpys*; *Ephx1*; *Fut8*; *Gbe1*; *Hmbs*; *Maoa*; *Maob*; *Mgst1*; *Pygl*; *Rdh3*; *Srd5a1*; *St3gal6*; *Ugt2a3*; *Ugt2b*; *Ugt2b10*; *Ugt2b17*; *Ugt2b36*; *Uros*
	Monosaccharide metabolism (enrichment score 2.84)	*Adh6*; *Agl*; *Aifm1*; *Aldh1a1*; *Aldh1b1*; *Atf4*; *Atp6v1a1*; *Bckdhb*; *Csnk1g3*; *Cyb5r3*; *Dhtkd1*; *Dio1*; *Dpys*; *Gbe1*; *Gm2a*; *Gpd2*; *Hmgcr*; *LOC685778*; *Maoa*; *Me1*; *Pck2*; *Pdhb*; *Pgd*; *Pgk1*; *Ppp1r3b*; *Prkaa1*; *Pygl*; *UAP1*; *GNPNAT1*; *Sat1*; *Slc16a1*; *Sod2*; *Sord*
	Pyruvate metabolism (enrichment score 2.23)	*Acat2*; *Acot12*; *Adh6*; *Aldh1b1*; *Atf4*; *Gpd2*; *LOC685778*; *Me1*; *Pck2*; *Pdhb*; *Pgk1*; *Slc16a1*
	Complement cascade and innate immunity (enrichment score 0.98)	*Baat*; *C2*; *Cabc1*; *Cadm1*; *Cfb*; *Dhcr24*; *Dnaja3*; *Erap1*; *F9*; *Ihh*; *LEAP2*; *LOC686548*; *Mbl1*; *Mbl2*; *Mif*; *Mst1*; *Nrep*; *RGD1560513*; *Serpina5*; *Sod2*
Repressed by leptin in UN offspring, induced by leptin in control offspring (Group2)	Metal ion binding (enrichment score 3.00)	*Aox1*; *Arsk*; *Atp2b1*; *Cdo1*; *Cnot6*; *Cyp2b3*; *Cyp3a3*; *Fancl*; *Gatad2b*; *Ireb2*; *ZZZ3*; *PHF20L1*; *Mdm4*; *Msl2l1*; *Nr5a2*; *Pcgf4*; *Pde8a*; *Phospho2*; *Prickle1*; *RGD1563633*; *Slc10a5*; *Slc30a7*; *Slc40a1*; *Thrb*; *Tmlhe*; *Trim33*; *Upb1*; *Zfp131*; *Zfp410*; *Zfp99*
Repressed by maternal undernutrition and by leptin treatment, but not by both combined (Groups 4 & 5)	MHC antigens and lymphocyte activation (enrichment score 2.43 → 4.00)	*Ccl5*; *Ccrl2*; *Cd69*; *Cd8a*; *Coro1a*; *Ctsl1*; *Fas*; *Gpsm3*; *Gstp1*; *H2-M3*; *Icam2*; *Igh-1a*; *Il1b*; *Itgb2*; *Klrd1*; *RGD1561628*; *Msh2*; *P2ry14*; *SH2D1A*; *Rpl9*; *RT1-149*; *RT1-A2*; *RT1-A3*; *RT1-Ba*; *RT1-CE15*; *RT1-CE7*; *RT1-M6-2*; *Sart1*; *Spn*; *Tap2*; *Tnfaip8l2*; *Tnfsf13*; *Unc13d*; *Unc93b1*; *Vcam1*; *Wdr46*; *Xcl1*
	Ribosomal biogenesis/translational elongation (enrichment score 2.27)	*Anxa1; Bloc1s2; Cpsf3; Epb4.1 l1; Gtf2b; Hnrpab; RPL22L2; Mrps25; Nufip1; Nup210; Polr2h; Polr2i; Polr2j; Pop4; RGD1559639; RGD1559951; Rnasen; Rpl17; Rpl18a; Rpl9; Rps14; Rps16; Rps17; Rps27; Sart1; Snrp1c; Tdrd3; Tubb5; Utp14a; Uxt; Wdr46*
Induced by the combination of maternal undernutrition and leptin treatment, but not by either factor alone (Group 6)	Ribosomal biogenesis/nucleolus (enrichment score 4.87)	*Agxt*; *Cct2*; *Cdc25a*; *Cdk105*; *Colq*; *Copb1*; *Dctn6*; *Epb4.1 l1*; *Fabp2*; *Ftl1*; *Gabarap*; *Gtf2a2*; *Gys2*; *Hnrnpa1*; *Hspb1*; *Imp3*; *Krt10*; *RPL22L1*; *LOC364236*; *RGD1563484*; *RPS7*; *RGD1564519*; *RGD1564606*; *POLR2E*; *Mapre3*; *Med6*; *Mettl11a*; *MGC114381*; *Mrpl49*; *Myo5b*; *Myrip*; *Nbn*; *Pola2*; *Psma6*; *Psma7*; *Psmb2*; *Ptk2*; *Ranbp1*; *Rassf5*; *WDR83*; *RGD1559574*; *RGD1559846*; *RGD1561086*; *RPS19L1*; *WTAP*; *RGD1565170*; *ZCCHC17*; *RGD1566137*; *RGD1566326*; *Rpain*; *Rpl27*; *Rpl35a*; *Rsl1d1*; *Sfrs2*; *Sqstm1*; *Tp53*; *Tuba4a*; *Vps33b*
	Cell cycle/Mitosis (enrichment score 1.70)	*Cdc25a*; *Gadd45gip1*; *Mapre3*; *MGC114381*; *Nbn*; *Psma6*; *Psma7*; *Psmb2*; *Ranbp1*; *Rgs10*; *Tp53*; *Usp16*

Note that individual genes may be annotated within several different functional clusters. Full DAVID output for each of the six groups is given in Additional file 3: Table S3.

(a) A series of metabolic and regulatory pathways were significantly over-represented in groups 1 and 3 (i.e. upregulated in AD/Lep and UN/Sal livers), particularly involving mitochondrially targeted genes. The observed shifts are consistent with thrifty metabolic reprogramming in these cohorts, and included several key enzymes involved in glycogenolysis and glycolysis, such as glygogen debranching enzyme (*Agl*), glycogen phosphorylase (*Pygl*), phosphoglycerate kinase (*Pgk*) and two subunits of pyruvate dehydrogenase (*Pdhb* and *LOC685778*). Also upregulated were important enzymes for amino acid and nitrogen metabolism, including glutamate dehydrogenase (*Glud*) and ornithine transcarbamoylase (*Otc*); lipid metabolism including glycerol phosphate dehydrogenase (*Gpd2*), long-chain fatty acid-CoA ligases (*Acsl3* and *Acsl5*) and acetyl-CoA acetyltransferase (*Acat2*); and oxidative stress genes including phosphogluconate dehydrogenase (*Pgd*) and mitochondrial outer membrane glutathione S-transferase (*Mgst1*). Consistent with the latter, although it did not fall into any of the annotated functional groups in the DAVID analysis, carbonic anhydrase 3 (*Ca3*) was also upregulated in AD/Lep and UN/Sal livers.

(b) Immune-related pathways showed a concerted pattern of change across the data set. Groups 4 and 5 (i.e. downregulated in AD/Lep and UN/Sal livers) were enriched for genes involved in the adaptive immune response, antigen presentation and lymphocyte activation. Particularly striking was a strong downregulation of MHC genes (~4-8 fold change of several class I genes, but also some class Ib and class II genes), and also a slight downregulation of the *Tap2* peptide transporter necessary for antigen presentation by class I molecules. In this light it is interesting to note that groups 1-3 (with the opposite expression pattern) showed a trend towards enrichment for genes involved in the complement cascade and innate immunity such as complement gene *C2*. However, this functional group was not statistically significant (enrichment score of 0.98, corresponding to p-value = 0.10). We note that alpha-fetoprotein (*Afp*), a specific marker for liver inflammation also falls into group 3, although it was not annotated as inflammation-related in the DAVID analysis.

(c) Pathways associated with ribosome biogenesis and function were significantly over-represented in group 6 and (to a lesser extent) groups 4 and 5. Finally, mitotic cell cycle genes showed weak over-represention in group 6. The significance level of this was borderline, with an enrichment score of 1.70 (p-value = 0.02).

Collectively, these obervations indicate that there is a programmed thrifty liver phenotype in AD/Lep and UN/Sal livers that includes increased glycogenolysis, an increase in protein breakdown and fat synthesis, and a switch away from adaptive immune function towards innate immunity/inflammation, together with increased oxidative stress within the cell.

### “Healthily thrifty” versus “unhealthily thrifty”: is there a difference?

Our finding that both AD/Lep and UN/Sal treatments appear to have similar transcriptional effects was unexpected, since in female rats only UN/Sal/HF develops metabolic syndrome while AD/Lep/HF does not. We therefore considered it possible that AD/Lep might represent a state of “healthy thriftiness”–i.e. a thrifty metabolism that is nevertheless under control and able to cope with a high fat diet without developing obesity; and that UN/Sal might represent “unhealthy thriftiness”–i.e. a thrifty metabolism that lacks safeguards and runs out of control when confronted with a high fat diet.

To test this possibility, we carried out a pairwise comparison of the AD/Lep/Chow and UN/Sal/Chow cohorts to see if there were any expression differences which might explain why the latter is predisposed to metabolic syndrome. However, we found no genes that were significantly differentially expressed (FDR p-value < 0.05) between these two cohorts, suggesting that the difference is one of degree rather than kind. Consistent with this, of 61 genes significantly differentially expressed (FDR p-value < 0.05) between UN/Sal/Chow and the control AD/Sal/Chow cohort, 58 showed the same direction of change in AD/Lep/Chow.

### Is thriftiness a consequence of growth restriction rather than leptin signalling *per se*?

It is challenging to explain why the UN/Sal and AD/Lep cohorts both show a thrifty transcriptional profile, given that postnatal leptin administration in the latter case will mimic excess nutrition rather than undernutrition. One possible explanation is that the dose/response curve for leptin is intrinsically bathtub-shaped, with adverse effects mediated both by excess and by insufficient leptin stimulation. This would be consistent with work showing that metabolic syndrome can be triggered by maternal overnutrition as well as maternal undernutrition [21]. However, an alternative and in our view more attractive explanation is that the commonalities in expression profiles for these cohorts reflect a shared early life history of growth retardation. In the case of the UN/Sal cohorts, the growth retardation is a direct consequence of the maternal undernutrition, while in the case of AD/Lep cohorts it occurs postnatally and is a secondary consequence of the leptin treatment.

**Figure 2:**
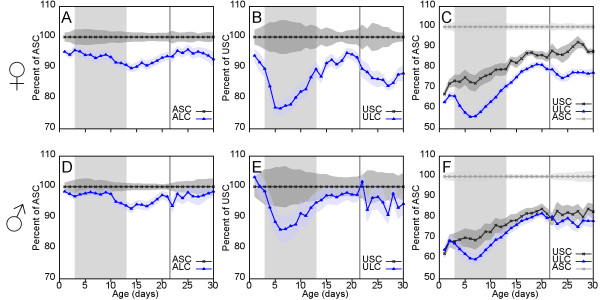
**Growth data for selected treatment cohorts and controls from postnatal days 1 to 30.** Leptin treatment (d3-13) is indicated by a shaded background. Weaning at day 22 is indicated with a vertical line. Plotted values indicate the average weight for each cohort as a percentage of control values: surrounding shaded areas show +/− s.e.m. at each age. **A**, **D**: Values for AD/Lep/Chow (blue) relative to AD/Sal/Chow (black) for females **(A)** and males **(D)** demonstrate a significant growth restriction in both sexes during leptin treatment. **B**, **E**: Values for UN/Lep/Chow (blue) relative to UN/Sal/Chow (black) for females **(B)** and males **(E)** demonstrate that in offspring of undernourished mothers there is an even more profound growth restriction during leptin treatment. Importantly, in females, but not in males, there is a pronounced fallback in body mass relative to control during and immediately following weaning (see text for discussion). **C**, **F**: Values for UN/Sal/Chow and UN/Lep/Chow relative to AD/Sal/Chow for females **(C)** and males **(F)**. The postweaning fallback in UN/Lep/Chow females is also seen when measured relative to AD/Sal/Chow, therefore it is not simply a consequence of catch-up growth in UN/Sal/Chow.

Figure 2A shows that while UN/Sal pups are severely growth restricted *in utero* as a consequence of the maternal undernutrition, AD/Lep pups also show a significant restriction in neonatal growth rate during the period of treatment (see Additional file 4: Table S4 for raw data). Although transient, the growth deficit is sufficient to induce a 5.9 percentage point drop in body mass relative to control, with AD/Lep body mass being 95.7% of AD/Sal at day 3, and 89.8% of AD/Sal at day 14. Other studies have shown that growth restriction in leptin-treated neonates is mediated by non-hepatic effects of leptin, particularly increased thermogenesis [22]. In the female experimental series analysed here, although the leptin-treated animals showed no decrease in food intake relative to saline-treated animals during the neonatal period and are thus not hypophagic [17], nevertheless it indicates that they have a negative energy balance given their increased energetic requirements and fail to take in sufficient nutrition to maintain a normal growth rate.

### The leptin rescue paradox: a conflict between thriftiness and set point programming

If maternal undernutrition and postnatal leptin treatment can both reprogramme liver gene expression to be thrifty, why then does the combination of both not do so? One possibility is that the postnatal leptin treatment might expunge the programme set during fetal life and then somehow fail to impose the postnatal programming seen in AD/Lep. Figure 2B shows that the postnatal leptin administration has the same growth-retarding effects in UN/Lep as in AD/Lep, rendering this hypothesis unlikely. In fact, the degree of growth suppression is considerably greater in the UN group, suggesting that the effect of leptin is potentiated by the prior starvation episode. If the growth restriction seen in AD/Lep is sufficient to trigger hepatic reprogramming, then it is reasonable to assume that the even greater effect in UN/Lep should also leave the liver in a thrifty state.

Figure 2B also shows that there is a significant drop in relative growth of the UN/Lep cohorts around weaning, which first becomes visible in the 1-2 days immediately prior to separation from the dam (i.e. when the pups begin to self-wean), and continues throughout the following week. This juncture marks the period in which pups take over full responsibility for their own metabolic balance between intake and expenditure, rather than it being partly controlled by maternal milk availability and composition. This postweaning drop in the UN/Lep cohort is not simply due to increased catch-up growth in UN/Sal, but reflects a genuine drop in weight relative to AD/Sal (Figure 2C). These observations support an explanation for the apparent paradox in which the two growth restriction episodes (both fetal and postnatal) in the UN/Lep cohorts do lead to thrifty metabolic programming in the livers of this group, but this is masked by non-hepatic programming effects of leptin on food intake and energy expenditure.

### Re-interpreting existing male growth data in the light of the female transcriptional findings

**Figure 3:**
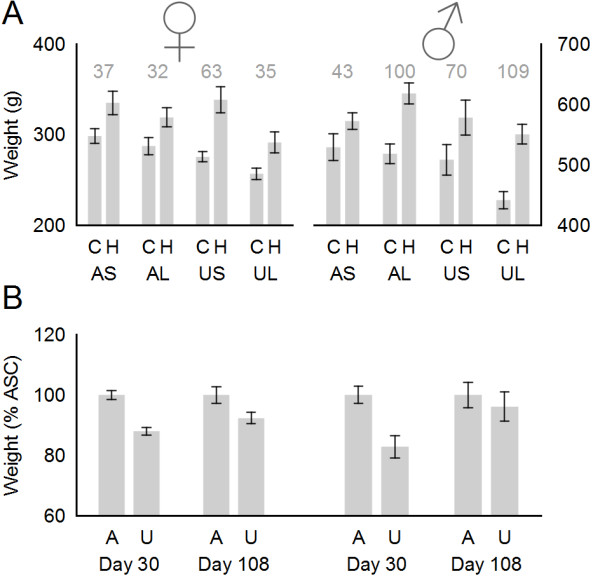
**Comparison of selected male and female growth data. A**: Bar chart showing average weight in g +/− s.e.m. for all cohorts at day 108. Numbers in grey indicate the absolute weight gain on HF diet relative to chow diet for the AD/Sal, AD/Lep, UN/Sal and UN/Lep groups. **B**: Bar chart showing weight of UN/Sal/Chow (U) relative to AD/Sal/Chow (A) at day 30 and day 108. Left = females, right = males.

In the light of the above findings, we also re-examined the growth data from our previously published experiment on male rats ( [18] and see also Additional file 4: Table S4 for raw data). Females and males show considerable differences in their programmed growth responses to postnatal leptin administration as early as weaning, where in males there is much less postweaning fallback in the UN/Lep cohort (Figure 2D-F). Responses also differ greatly between adult males and females. Figure 3A shows the absolute weight gain on HF relative to normal chow diet for each group of males and females. For AD/Sal, both males and females gain comparable amounts on a HF diet (37 g and 43 g respectively). Similarly, for UN/Sal, both males and females gain a larger amount on the HF diet (63 g and 70 g respectively). For AD/Lep, females gain 32 g on the HF diet while males gain 100 g. Finally, for UN/Lep, females show an efficient rescue, bringing the weight gain back down to 35 g (from 63 in UN/Sal): by contrast males show no rescue, instead gaining 109 g, the highest of any treatment cohort.

The pathogenic programming effects of maternal undernutrition are also more severe in males than females, in that they are not restricted to males on the HF diet. Figure 3C shows the weight of UN/Sal/Chow cohorts relative to control AD/Sal/Chow for males and females at day 30 and at the end of the experimental period. Females and males both showed a substantial growth restriction (see also Figure 2C, F) that is somewhat more severe in males (82.9% ±3.7%) than females (88.0% ± 1.3%) at day 30. These males then showed a continued slow weight gain throughout life relative to control, reaching 96.1 ± 4.8% of control by day 108–note that this was the end of the male experimental series. In contrast, the females maintained a stable weight relative to control from day 30 onwards (92.3% ± 1.3% on day 108, and 89.8% ± 2.4% on day 171–the end of the female experiment).

Collectively, these results suggest that while the pathogenic “thrift-inducing” effects of early growth restriction are shared between males and females, the non-hepatic mechanisms underlying the leptin rescue in UN/Lep cohorts may be less effective (or even absent) in males compared to females. In the Discussion (below) we propose a revised and extended model for developmental programming, which reconciles the hitherto-conflicting male and female growth data.

### Genes associated with the prevention of metabolic syndrome by leptin treatment in the UN/Lep/HF cohort

Although the AD/Lep cohorts globally show many of the same metabolic and transcriptional changes as the UN/Sal cohorts, nevertheless in females metabolic syndrome is only triggered in UN/Sal/HF cohort and is prevented by postnatal leptin treatment (“leptin rescue”) in UN/Lep/HF. It is important to determine the hepatic transcriptional changes associated with the leptin rescue, since these may be useful biomarkers for metabolic syndrome and/or for the predisposed state seen in UN/Sal/Chow. Genes of interest in this regard are those that show a significant perturbation in expression associated with metabolic syndrome, which is exacerbated by maternal undernutrition and rescued by postnatal leptin treatment. To identify these genes, we took the initial set of 2221 genes showing at least some significant change in the one-way ANOVA and imposed three further filters:

• Maternal diet and leptin treatment both called as significant, either individually or as part of an interaction term. 1010 genes passed this filter; the 949 genes with a significant AB interaction plus a further 61 genes where both factors were individually significant but the AB interaction term was not significant.

• At least a 1.25 fold change in expression level between the affected UN/Sal/HF cohort and the control AD/Sal/Chow cohort. 177 genes passed this filter.

• Gene expression levels in the UN/Sal/HF cohort with metabolic syndrome must fall at an extreme (highest or lowest expression amongst all 8 experimental cohorts). This yielded a final set of 98 genes, 56 of which were upregulated in metabolic syndrome and 42 downregulated (Table 3 and Additional file 5: Table S5).

**Table 3:** Genes which show a significant interaction between maternal nutritional status and postnatal leptin treatment effects, an expression change of at least 1.25 fold in the UN/Sal/HF cohort (with metabolic syndrome), and where this cohort is an outlier from the other experimental groups

**Symbol**	**Fold change in USC**	**Fold change in USH**	**Significant factors**	**Entrez ID**	**Definition**
**Ifit1**	**2.95**	**3.52**	**AB**	**56824**	**Rattus norvegicus interferon-induced protein with tetratricopeptide repeats 1 (Ifit1), mRNA.**
**Ppp1r3b**	**1.46**	**2.40**	**AB, C**	**192280**	**Rattus norvegicus protein phosphatase 1, regulatory (inhibitor) subunit 3B (Ppp1r3b), mRNA.**
**Ca3**	**1.29**	**2.38**	**AB, C**	**54232**	**Rattus norvegicus carbonic anhydrase 3 (Ca3), mRNA.**
**Stac3**	**1.27**	**2.35**	**AB, C**	**362895**	**PREDICTED: Rattus norvegicus SH3 and cysteine rich domain 3 (predicted) (Stac3_predicted), mRNA.**
**TUBB2A**	**1.79**	**2.33**	**AB, BC**	**498736**	**PREDICTED: Rattus norvegicus similar to tubulin, beta 2 (TUBB2A), mRNA.**
**LEAP2**	**1.52**	**2.29**	**AB, C**	**497901**	**PREDICTED: Rattus norvegicus similar to Liver-expressed antimicrobial peptide 2 precursor (LEAP-2) (LEAP2), mRNA.**
**Slc34a2**	**1.45**	**1.88**	**AB, C**	**84395**	**Rattus norvegicus solute carrier family 34 (sodium phosphate), member 2 (Slc34a2), mRNA.**
Pgd	1.24	1.77	AB	362660	PREDICTED: Rattus norvegicus phosphogluconate dehydrogenase (Pgd), mRNA.
**Afp**	**1.33**	**1.76**	**AB, C**	**24177**	**Rattus norvegicus alpha-fetoprotein (Afp), mRNA.**
VNN3	1.17	1.70	AB, C	498992	PREDICTED: Rattus norvegicus similar to Vanin-3 (predicted) (VNN3_predicted), mRNA.
**Avpr1a**	**1.42**	**1.70**	**AB, C**	**25107**	**Rattus norvegicus arginine vasopressin receptor 1A (Avpr1a), mRNA.**
Laptm4b	0.94	1.68	AB	315047	Rattus norvegicus lysosomal-associated protein transmembrane 4B (Laptm4b), mRNA.
Igf2bp3	1.23	1.67	AB, C	312320	PREDICTED: Rattus norvegicus insulin-like growth factor 2, binding protein 3 (Igf2bp3), mRNA.
Aldh1b1	1.08	1.64	AB, C	298079	Rattus norvegicus aldehyde dehydrogenase 1 family, member B1 (Aldh1b1), nuclear gene encoding mitochondrial protein, mRNA.
**Amy1a**	**1.33**	**1.64**	**AB, C**	**24203**	**Rattus norvegicus amylase, alpha 1A (salivary) (Amy1a), mRNA.**
Aadac	1.19	1.62	AB, C	57300	Rattus norvegicus arylacetamide deacetylase (esterase) (Aadac), mRNA.
**Pipox**	**1.56**	**1.60**	**AB, BC**	**303272**	**Rattus norvegicus pipecolic acid oxidase (Pipox), mRNA.**
**Sez6**	**1.28**	**1.58**	**AB, C**	**192247**	**PREDICTED: Rattus norvegicus seizure related 6 homolog (mouse) (Sez6), mRNA.**
**Olr59**	**1.31**	**1.56**	**AB, C**	**170816**	**Rattus norvegicus olfactory receptor 59 (Olr59), mRNA.**
**RGD1563713**	**1.31**	**1.56**	**AB**	**316550**	**PREDICTED: Rattus norvegicus similar to Rab18 (RGD1563713), mRNA.**
**Gpd2**	**1.28**	**1.56**	**AB, C**	**25062**	**Rattus norvegicus glycerol-3-phosphate dehydrogenase 2, mitochondrial (Gpd2), nuclear gene encoding mitochondrial protein, mRNA.**
Bche	1.20	1.51	AB, C	65036	Rattus norvegicus butyrylcholinesterase (Bche), mRNA.
**Otc**	**1.32**	**1.51**	**AB, C**	**25611**	**Rattus norvegicus ornithine carbamoyltransferase (Otc), nuclear gene encoding mitochondrial protein, mRNA.**
Dnmt3b	0.95	1.51	AB, AC, BC	444985	Rattus norvegicus DNA methyltransferase 3B (Dnmt3b), mRNA.
**Clpx**	**1.35**	**1.50**	**AB, BC**	**300786**	**Rattus norvegicus ClpX caseinolytic peptidase X homolog (E. coli) (Clpx), mRNA.**
Slc7a7	1.03	1.49	AB, C	83509	Rattus norvegicus solute carrier family 7 (cationic amino acid transporter, y + system), member 7 (Slc7a7), mRNA.
Dpys	1.04	1.49	AB, C	65135	Rattus norvegicus dihydropyrimidinase (Dpys), mRNA.
**Fam82a**	**1.34**	**1.48**	**AB, C**	**313840**	**Rattus norvegicus family with sequence similarity 82, member A (Fam82a), mRNA.**
Slc16a1	1.15	1.41	AB	25027	Rattus norvegicus solute carrier family 16 (monocarboxylic acid transporters), member 1 (Slc16a1), mRNA.
**Adhfe1**	**1.32**	**1.40**	**AB, C**	**362474**	**Rattus norvegicus alcohol dehydrogenase, iron containing, 1 (Adhfe1), mRNA.**
Inhba	1.10	1.40	AB, BC	29200	Rattus norvegicus inhibin beta-A (Inhba), mRNA.
TMEM70	1.13	1.38	AB, C	500384	PREDICTED: Rattus norvegicus similar to RIKEN cDNA 2210416 J16 (predicted) (TMEM70_predicted), mRNA.
RGD1560797	1.08	1.38	A, BC	306115	PREDICTED: Rattus norvegicus similar to glyceraldehyde-3-phosphate dehydrogenase (predicted) (RGD1560797_predicted), mRNA.
RGD1310209	1.08	1.35	AB, C	362019	PREDICTED: Rattus norvegicus similar to KIAA1324 protein (predicted) (RGD1310209_predicted), mRNA.
Maob	1.24	1.34	AB	25750	Rattus norvegicus monoamine oxidase B (Maob), nuclear gene encoding mitochondrial protein, mRNA.
Lrp5	1.09	1.34	AB	293649	PREDICTED: Rattus norvegicus low density lipoprotein receptor-related protein 5 (predicted) (Lrp5_predicted), mRNA.
Klrg1	1.10	1.34	AB, C	58975	Rattus norvegicus killer cell lectin-like receptor subfamily G, member 1 (Klrg1), mRNA.
Atf4	1.24	1.33	AB	79255	Rattus norvegicus activating transcription factor 4 (tax-responsive enhancer element B67) (Atf4), mRNA.
Phf11	1.15	1.33	AB, C	361051	PREDICTED: Rattus norvegicus PHD finger protein 11 (predicted) (Phf11_predicted), mRNA.
Rora	1.25	1.33	AB	300807	PREDICTED: Rattus norvegicus RAR-related orphan receptor alpha (predicted) (Rora_predicted), mRNA.
Serpina3m	1.17	1.32	AB, C	299276	PREDICTED: Rattus norvegicus serine (or cysteine) proteinase inhibitor, clade A, member 3 M (Serpina3m), mRNA.
PLBD1	1.15	1.31	AB, C	297694	Rattus norvegicus similar to RIKEN cDNA 1100001H23 (PLBD1), mRNA.
ANGPTL3	1.17	1.31	AB, C	502970	PREDICTED: Rattus norvegicus similar to angiopoietin-related protein 3 (ANGPTL3), mRNA.
Agtr1a	1.02	1.30	AB	24180	Rattus norvegicus angiotensin II receptor, type 1 (AT1A) (Agtr1a), mRNA.
Apon	1.09	1.30	AB, C	304603	Rattus norvegicus apolipoprotein N (Apon), mRNA.
Lgals5	1.21	1.29	AB	25475	Rattus norvegicus lectin, galactose binding, soluble 5 (Lgals5), mRNA.
Cabc1	1.23	1.28	AB	360887	Rattus norvegicus chaperone, ABC1 activity of bc1 complex homolog (S. pombe) (Cabc1), nuclear gene encoding mitochondrial protein, mRNA.
Sdcbp	1.12	1.27	AB	83841	Rattus norvegicus syndecan binding protein (Sdcbp), mRNA.
CP	1.07	1.27	AB	294942	PREDICTED: Rattus norvegicus hypothetical CP (CP), mRNA.
Irf6	1.14	1.26	AB	364081	PREDICTED: Rattus norvegicus interferon regulatory factor 6 (predicted) (Irf6_predicted), mRNA.
Hsd17b13	1.07	1.26	AB, C	305150	PREDICTED: Rattus norvegicus hydroxysteroid (17-beta) dehydrogenase 13 (Hsd17b13), mRNA.
Mst1	1.17	1.26	AB	24566	Rattus norvegicus Macrophage stimulating 1 (hepatocyte growth factor-like) (Mst1), mRNA.
Btg1	1.13	1.25	AB, C	29618	Rattus norvegicus B-cell translocation gene 1, anti-proliferative (Btg1), mRNA.
MUT	1.19	1.25	AB	497857	PREDICTED: Rattus norvegicus similar to MYLE protein (Dexamethasone-induced protein) (predicted) (MUT_predicted), mRNA.
**Pxmp4**	**−1.38**	**−3.51**	**AB**	**282634**	**Rattus norvegicus peroxisomal membrane protein 4 (Pxmp4), mRNA.**
**Igfbp2**	**−2.11**	**−3.09**	**AB, C**	**25662**	**Rattus norvegicus insulin-like growth factor binding protein 2 (Igfbp2), mRNA.**
**Sds**	**−1.54**	**−2.49**	**AB**	**25044**	**Rattus norvegicus serine dehydratase (Sds), mRNA.**
**Tnfsf13**	**−2.22**	**−2.42**	**AB**	**287437**	**Rattus norvegicus tumor necrosis factor (ligand) superfamily, member 13 (Tnfsf13), mRNA.**
Hnrpab	−1.19	−2.09	AB, C	83498	Rattus norvegicus heterogeneous nuclear ribonucleoprotein A/B (Hnrpab), mRNA.
**Asl**	**−1.33**	**−2.07**	**AB, C**	**59085**	**Rattus norvegicus argininosuccinate lyase (Asl), mRNA.**
**Cd63**	**−1.36**	**−1.94**	**AB, C**	**29186**	**Rattus norvegicus CD63 antigen (Cd63), mRNA.**
**RGD1560687**	**−1.25**	**−1.85**	**AB, C**	**500804**	**PREDICTED: Rattus norvegicus similar to Ferritin light chain (Ferritin L subunit) (predicted) (RGD1560687_predicted), mRNA.**
**Trpm6**	**−1.27**	**−1.76**	**AB, C**	**293874**	**PREDICTED: Rattus norvegicus transient receptor potential cation channel, subfamily M, member 6 (predicted) (Trpm6_predicted), mRNA.**
**Hspb1**	**−1.34**	**−1.68**	**AB, C**	**24471**	**Rattus norvegicus heat shock 27 kDa protein 1 (Hspb1), mRNA.**
Ftl1	−1.11	−1.60	AB, C	29292	Rattus norvegicus ferritin light chain 1 (Ftl1), mRNA.
**Mgst2**	**−1.43**	**−1.54**	**AB, BC**	**295037**	**PREDICTED: Rattus norvegicus microsomal glutathione S-transferase 2 (predicted) (Mgst2_predicted), mRNA.**
Znf593	−1.19	−1.53	AB, C	298546	PREDICTED: Rattus norvegicus zinc finger protein 593 (predicted) (Znf593_predicted), mRNA.
**Ppcs**	**−1.43**	**−1.52**	**AB, C**	**298490**	**Rattus norvegicus phosphopantothenoylcysteine synthetase (Ppcs), mRNA.**
**Mthfd1**	**−1.38**	**−1.47**	**A, BC**	**64300**	**Rattus norvegicus methylenetetrahydrofolate dehydrogenase (NADP + dependent) 1, methenyltetrahydrofolate cyclohydrolase, formyltetrahydrofolate synthetase (Mthfd1), mRNA.**
**Pmm1**	**−1.26**	**−1.47**	**A, B, C**	**300089**	**Rattus norvegicus phosphomannomutase 1 (Pmm1), mRNA.**
**Aqp11**	**−1.35**	**−1.45**	**AB**	**286758**	**Rattus norvegicus aquaporin 11 (Aqp11), mRNA.**
**Csrp2**	**−1.34**	**−1.45**	**A, B, C**	**29317**	**Rattus norvegicus cysteine and glycine-rich protein 2 (Csrp2), mRNA.**
**Pla2g12a**	**−1.27**	**−1.44**	**AB, BC**	**362039**	**PREDICTED: Rattus norvegicus phospholipase A2, group XIIA (predicted) (Pla2g12a_predicted), mRNA.**
Tifa	−1.24	−1.42	AB, C	310877	Rattus norvegicus TRAF-interacting protein with forkhead-associated domain (Tifa), mRNA.
Hexb	−1.17	−1.41	AB, C	294673	Rattus norvegicus hexosaminidase B (Hexb), mRNA.
Rps14	−1.15	−1.41	AB, C	29284	Rattus norvegicus ribosomal protein S14 (Rps14), mRNA.
**Ier3**	**−1.38**	**−1.40**	**AB**	**294235**	**Rattus norvegicus immediate early response 3 (Ier3), mRNA.**
**Paics**	**−1.40**	**−1.40**	**AB**	**140946**	**Rattus norvegicus phosphoribosylaminoimidazole carboxylase, phosphoribosylaminoimidazole succinocarboxamide synthetase (Paics), mRNA.**
**Txnl5**	**−1.32**	**−1.38**	**AB**	**287474**	**PREDICTED: Rattus norvegicus thioredoxin-like 5 (predicted) (Txnl5_predicted), mRNA.**
**Pla1a**	**−1.35**	**−1.38**	**AB**	**85311**	**Rattus norvegicus phospholipase A1 member A (Pla1a), mRNA.**
Cyp8b1	−1.08	−1.37	AB, C	81924	Rattus norvegicus cytochrome P450, family 8, subfamily b, polypeptide 1 (Cyp8b1), mRNA.
**Creb3**	**−1.28**	**−1.36**	**AB, C**	**298400**	**Rattus norvegicus cAMP responsive element binding protein 3 (Creb3), mRNA.**
Cd320	−1.06	−1.36	AB, C	362851	PREDICTED: Rattus norvegicus CD320 antigen (Cd320), mRNA.
Nola2	−1.10	−1.34	AB, C	287273	PREDICTED: Rattus norvegicus nucleolar protein family A, member 2 (predicted) (Nola2_predicted), mRNA.
Sult1c1	−0.87	−1.32	AB	65185	Rattus norvegicus sulfotransferase family, cytosolic, 1C, member 1 (Sult1c1), mRNA.
Slc11a2	−1.16	−1.32	AB, BC	25715	Rattus norvegicus solute carrier family 11 (proton-coupled divalent metal ion transporters), member 2 (Slc11a2), mRNA.
Btbd9	−1.19	−1.31	A, BC	294318	Rattus norvegicus BTB (POZ) domain containing 9 (Btbd9), mRNA.
Htatip2	−1.20	−1.30	AB, C	292935	PREDICTED: Rattus norvegicus HIV-1 tat interactive protein 2, homolog (human) (predicted) (Htatip2_predicted), mRNA.
Ctsl1	−1.09	−1.29	AB, C	25697	Rattus norvegicus cathepsin L1 (Ctsl1), mRNA.
Psmb3	−1.14	−1.29	AB, C	29676	Rattus norvegicus proteasome (prosome, macropain) subunit, beta type 3 (Psmb3), mRNA.
Sat2	−1.16	−1.29	AB	360547	PREDICTED: Rattus norvegicus spermidine/spermine N1-acetyl transferase 2 (predicted) (Sat2_predicted), mRNA.
Gtf2b	−1.15	−1.29	AB, C	81673	Rattus norvegicus general transcription factor IIB (Gtf2b), mRNA.
Eppb9	−1.10	−1.26	AB, BC	287383	PREDICTED: Rattus norvegicus endothelial precursor protein B9 (predicted) (Eppb9_predicted), mRNA.
Sdf2	−1.05	−1.26	AB	287470	PREDICTED: Rattus norvegicus stromal cell derived factor 2 (predicted) (Sdf2_predicted), mRNA.
Wdr45	−1.17	−1.26	AB, C	302559	Rattus norvegicus WD repeat domain 45 (Wdr45), mRNA.
Psma5	−1.20	−1.26	AB, C	29672	Rattus norvegicus proteasome (prosome, macropain) subunit, alpha type 5 (Psma5), mRNA.

Bold indicates genes where the expression change in the UN/Sal/Chow cohort (predisposed to metabolic syndrome) is also at least 1.25 fold, indicating that precursor changes are occurring in this group also. Additional file 5: Table S5 is an expanded version of this table containing further annotation relating to gene ontology, gene function and known human diseases related to these genes.

DAVID analysis of the resulting gene list (see Additional file 5: Table S5) reveals significant deregulation of carbohydrate metabolic pathways (enrichment factor 2.939, p-value = 0.00115), steroid response genes (enrichment factor 1.59, p-value = 0.0256) and mitochondrially-targeted genes (enrichment factor 1.53, p-value = 0.0298).

There was no significant enrichment for functional terms relating to immune function in this list since the significant downregulation of MHC class I was more pronounced in AD/Lep cohorts than in UN/Sal cohorts and hence these were excluded by the final filter (the same applies to the upregulation of complement gene C2). It is at present unclear why postnatal leptin treatment should have such a marked effect on immune parameters.

### Precursor changes in chow-fed cohorts may be associated with disease predisposition

Of the four chow-fed cohorts, none directly shows metabolic syndrome, however the UN/Sal/Chow cohort is predisposed to metabolic syndrome and will suffer from it if fed a HF diet. We therefore looked specifically within the chow-fed cohorts to determine whether any of the 98 genes associated with metabolic syndrome (and rescued by leptin treatment) showed precursor expression changes that might be involved in susceptibility to or initiation of metabolic syndrome. In all, 41/98 of these genes also showed at least a 1.25 fold expression change in the UN/Sal/Chow cohort relative to the control AD/Sal/Chow cohort (highlighted in bold in Table 3). For these precursor changes, DAVID analysis did not show any enrichment for mitochondrially-targeted genes or for steroid response genes, however the annotation group relating to carbohydrate metabolism remained significant (enrichment factor 2.71, p-value = 0.002).

## Discussion

In this study, we present a whole-genome fully factorial examination of the transcriptional consequences of the interactions between maternal diet, leptin treatment, postweaning diet and metabolic syndrome in a rat model of developmental programming.

Our data confirm the prior observation of “leptin reversal”, in that of the genes regulated by postnatal leptin treatment, the vast majority show the opposite direction of leptin-induced change in pups born to undernourished mothers, when compared to those born to normally-nourished mothers. Strikingly, it is not simply that maternal undernutrition reverses the direction of the leptin response: rather it appears that both maternal undernutrition and postnatal leptin treatment independently switch the liver into an altered state. Paradoxically, the combination of both factors abolishes this effect and switches the liver back into a state more closely resembling the control (untreated) cohorts. This pattern is seen in both the transcriptional data and in key phenotypic parameters relating to metabolic status.

### A revised model for developmental programming of obesity

Based on the transcriptional findings reported above and on our re-examination of existing growth data for both male and female offspring, we propose a revised model of developmental programming by maternal undernutrition and postnatal leptin treatment. In this new model, there are two competing programmes that can be established quasi-independently. The first programme is a thrifty hepatic metabolic programme induced by early life growth restriction, governing the efficiency with which fuel is used by the body. Importantly, in the AD/Lep cohorts the thrifty programming is invoked even in the presence of a supraphysiological leptin stimulus, suggesting that leptin itself is not the predominant signal involved in programming of hepatic metabolic efficiency. The second programme is a homeostatic set point programme governing body composition that is established in response to leptin stimulation during early neonatal life. The calibration of the set point (either lean or normal body composition) is determined by the body’s composition at the time of leptin stimulation, while the robustness with which the programme is “locked in” depends on the level of the stimulus. Given the known effects of leptin on appetite and locomotor activity, it is likely that this second programme is mediated at least in part by centrally controlled adjustment of calorie intake and expenditure, however we cannot rule out effects of leptin in other non-hepatic tissues.

Our model is based on the emerging view that during development leptin functions as an anti-starvation signal, as distinct from its adult role as a fatness and satiety signal [23]. During early development, in normally nourished animals, leptin levels are high (known as the “leptin surge”) and largely independent of fat mass. As development proceeds, leptin levels drop to a lower level governed dually by overall fat mass and satiety [24-26]. Early life undernutrition delays or abolishes the leptin surge [27,28], and nutritional recovery restores it [29]–in the latter study, higher leptin levels were observed in “recovered” infants than in control infants, which we argue may represent a delayed developmental surge rather than a simple restoration of leptin secretion following fat gain.

We hypothesise that the function of neonatal leptin signalling is to train the homeostatic set point mechanisms to recognise and subsequently maintain an appropriate body fat composition, and that this pattern (surge followed by drop) represents the calibration mechanism for the feedback loop. The peak of the leptin surge triggers developments underpinning set point programming, while the leptin levels immediately subsequent to the surge set the baseline which will subsequently be defended by the newly-calibrated set point program. If the individual is normal weight at the time of the leptin surge, a normal calibration is established and will subsequently be maintained. Under starvation conditions the calibration process would normally be delayed by mechanisms including lower overall leptin secretion and also competition by excess soluble leptin receptor [29]–however if exogenous leptin is administered, the combination of a leptin surge with an underweight body composition means that an unusually lean calibration is established and will subsequently be maintained. Conversely, if the individual is overweight at the time of the leptin surge, the calibration is set inappropriately high.

### Interpreting the differences between male and female responses to early programming

Our existing growth data for male rats suggests that the homeostatic set point mechanism is less efficient in males than in females. Interestingly, in human babies there is a sex difference in favour of higher leptin concentrations in female newborns [26], which is consistent with our above hypothesis about the function of the leptin surge in set point calibration. Incorporating this final factor leads to the unified model described in Table 4, which summarises the key elements of our new model and how it relates to the observed phenotypes in both sexes. Briefly, the first, hepatic element of the programme (i.e. thrifty use of fuel) is induced by early life growth restriction irrespective of the reason for that growth restriction, and as such all except the AD/Sal cohorts are programmed to be thrifty. The second, leptin-mediated element of the programme (i.e. the homeostatic set point) varies considerably between cohorts. The AD/Sal and AD/Lep cohorts have a normal body composition at the time of leptin stimulation and thus are calibrated to a normal set point. The UN/Lep cohorts have a very lean body composition at the time of leptin stimulation and thus are calibrated to a lean set point. The UN/Sal cohorts also have a lean body composition postnatally and are therefore calibrated to a lean set point, but since there is no exogenous leptin given and endogenous levels are low, the calibration is only weakly enforced. Consequently, in the UN/Lep cohort the set point mechanisms are strong enough to over-ride the metabolic programme and enforce a lean body composition, masking the thrifty hepatic metabolic programming, while in the UN/Sal cohort they cannot do so. In males the hepatic element of the programme is maintained, while the set point enforcement is weaker for unknown reasons.

**Table 4:** Unified model for how the competing hepatic thriftiness and non-hepatic set point programmes interact to produce the observed phenotypes in males and females

	**Cohort**	**Metabolism**^**1**^	**Set point calibration**^**2**^	**Set point enforcement**^**3**^	**Phenotypic consequences**
♀	**AD/Sal**	**Normal**	**Normal**	**Strong** (female default)	Baseline for chow and high fat diet regimes in females.
	**AD/Lep**	**Thrifty**	**Normal**	**Strong** (female default)	Normal set point calibration is enforced and overrides the thrifty metabolism. Weight remains comparable to AD/Sal females on both low and high fat diets despite thrifty liver biochemistry.
	**UN/Sal**	**Thrifty**	**Lean**	**Impaired** (due to insufficient leptin availability)	Set point enforcement is impaired and consequently unable to compensate for a thrifty metabolism when fed a HF diet. UN/Sal/HF females become morbidly obese and suffer metabolic syndrome.
	**UN/Lep**	**Thrifty**	**Lean**	**Strong** (restored to female default level)	A well-enforced “lean” set point in rescued females compensates for the thrifty metabolism, enforcing a lean body composition in UN/Lep/Chow and keeping weight gain down to normal levels in UN/Lep/HF.
♂	**AD/Sal**	**Normal**	**Normal**	**Weak** (male default)	Baseline for chow and high fat diet regimes in males.
	**AD/Lep**	**Thrifty**	**Normal**	**Weak** (male default)	As with UN/Sal animals (male and female), set point enforcement is weak and cannot compensate for the thrifty metabolism. The AD/Lep HF phenotype is more severe than UN/Sal/HF because the set point is higher.
	**UN/Sal**	**Thrifty**	**Lean**	**Severely impaired** (weak male default is lowered further)	Set point enforcement is severely impaired and thus UN/Sal males cannot maintain a steady body composition even on a normal chow diet. However, the set point is still lean and so the phenotype on HF diet is less severe than AD/Lep males.
	**UN/Lep**	**Thrifty**	**Lean**	**Weak** (restored to male default level)	Combined programme is similar to UN/Sal females. Rescued males maintain a steady body composition on a normal chow diet but show elevated weight gain on HF diet.

^1^The thrifty metabolic programme is induced by periods of restricted growth, whether due to maternal undernutrition or postnatal leptin treatment, and is consequently present in all except AD/Sal cohorts.

^2^The level of the set point is established by the body composition at the time of the neonatal leptin surge, and is consequently normal for AD cohorts and lean for UN cohorts.

^3^The strength with which the set point calibration is enforced depends on the levels of leptin at the time of calibration. Set point enforcement is weaker in males than in females for unknown reasons.

The new model thus explains four key puzzles concerning various features of the programmed phenotypes in males and females of this rat model, namely;

(1) In females AD/Lep and UN/Sal both induce transcriptional changes related to fatty liver disease, but the combination of both in UN/Lep does not (Figure 1 and Results *passim*).

(2) AD/Lep treatment is obesogenic in males but not in females (Figure 3A).

(3) Neonatal leptin treatment protects against metabolic syndrome in UN females on a high fat postweaning diet, but is much less effective in males (Figure 3A).

(4) UN/Sal/Chow females maintain a constant weight relative to AD/Sal/Chow controls from weaning onwards, whereas UN/Sal/Chow males shows a lifelong slow increase relative to AD/Sal/Chow controls (Figure 3B).

### Significance of the “calibration” model for postnatal leptin signalling

Potentially this view of postnatal leptin signalling as a training/calibration mechanism is also applicable in wider contexts. For example, maternal overnutrition (as well as undernutrition) can also programme metabolic syndrome and obesity in offspring [21]. Under our model, this is explained by the offspring being fatter at the time of the postnatal leptin training signal. The set point is consequently established at a higher level, leading these individuals to maintain and defend an inappropriately high body fat composition.

Similarly, this model could explain the differences in outcome (seen in both human and animal models) between neonates exhibiting rapid catch-up growth after starvation and those with a slower return to normal weight [30,31]. Given that rapid weight recovery leads disproportionately to fat gain rather than lean tissue gain, it will skew the overall body composition to a higher fat percentage, which will subsequently be “locked in” once the leptin signalling reaches a high enough level to trigger the establishment of a body composition set point. Consistent with this, in a rat model, rapid catch up growth leads to leptin resistance in adulthood–the reduced sensitivity will consequently lead these animals to self-regulate to a higher plasma leptin level and a higher body fat content. Conversely, slower catch up growth does not have the same effect on the leptin feedback loop [32,33].

Of key interest for future research will be whether this post-natal calibration of the body’s set point is a one-off event during a specific developmental window, or whether it can be re-triggered by a leptin surge (endogenous or exogenous) at later ages. One can envisage a “ratchet” mechanism for morbid obesity whereby weight gain beyond a given threshold triggers a re-calibration and establishment of a “new normal”, making subsequent weight loss harder. Conversely, if “surge” levels of leptin were administered following a period of weight loss, would it then be able to re-establish a healthier set point for the patient?

### A thrifty liver is an immunosuppressed, inflamed liver

In functional terms, there are two core axes affected by the phenotypic switch in the liver, namely metabolism-and immune-related functions. The metabolism-related changes include upregulation of multiple genes concerned with mitochondrial function, glycolysis, gluconeogenesis and carbohydrate metabolism. This is associated with increased total body fat percentage, increased circulating fasting leptin levels, and increased insulin/C-peptide levels.

The second functional cluster of expression changes observed relates to immune function, with a concerted downregulation of MHC genes (predominantly class I genes, but also some class Ib and class II genes) and the *Tap2* peptide transporter necessary for antigen presentation by class I molecules. Concurrent with this downregulation of adaptive immunity, there is a trend towards upregulation of the innate immune system, including both generic inflammatory mediators such as complement and also liver-specific markers such as alpha-fetoprotein. These changes are consistent with a model of non-alcoholic fatty liver disease as a chronic low-grade inflammatory process [34].

Concurrent with the pro-inflammatory changes observed, the effects on the adaptive immune system are interesting in relation to the immunological effects of perinatal programming, in particular the increased mortality from infectious disease in *in utero*-deprived children [13,14]. Mechanistically, there is a plausible link between the metabolic phenotype and the immune system changes in that excess saturated fatty acids are known to exert an immunosuppressive effect via reduced antigen presentation by MHC class I molecules [35-37]. Potentially therefore this rat model may in future serve as a model for immune system development in malnourished children, as well as for metabolic disease.

It is intriguing to note that these transcriptional changes–both those reflecting increased inflammation and those reflecting decreased antigen presentation–are not only seen in the UN/Sal/HF cohort, but are consistent across all AD/Lep and UN/Sal cohorts irrespective of postweaning diet. This suggests that the immune changes are directly associated with the thrifty phenotype itself rather than with metabolic syndrome. An important avenue for future research will therefore be to examine immune parameters at early timepoints in programmed thrifty and non-thrifty animals to confirm whether the immune changes do indeed precede the development of clinical disease, and what impact this has for our understanding of the pathogenesis of obesity.

### Do variations in hepatic thriftiness affect predisposition to metabolic syndrome in a nutritionally rich environment?

In the female experiment analysed here, despite the fact that both AD/Lep and UN/Sal provoke the same thriftiness phenotypic switch within the liver, it is only the UN/Sal rats that subsequently develop the full hallmarks of metabolic syndrome when fed a high fat diet. As we argue above, we believe this is most likely due to non-hepatic effects of leptin in establishing and maintaining a set point for body composition, however it is also plausible that there may be varying degrees of hepatic thriftiness depending on the nature and intensity of the stimulus provoking the programmed changes.

Therefore, we looked in detail at genes most strongly associated with pathological status (i.e. genes where UN/Sal/HF is an outlier from all other cohorts) and which were also affected by the maternal diet/leptin interaction. These are the genes most strongly associated with the induction of metabolic syndrome by maternal undernutrition and its subsequent rescue by leptin treatment. These too showed disturbances of carbohydrate metabolism and an increase in gluconeogenesis. Strikingly, around a quarter of this final set of genes showed precursor changes in the UN/Sal/Chow cohort, which potentially represent pre-pathogenic events that predispose to metabolic syndrome even among rats on a normal diet. Further investigation of this cohort could potentially indicate biomarkers for those at *risk* of metabolic syndrome, allowing targeted intervention and/or advice to be given before the onset of pathological change. Conversely, investigation of the non-hepatic effects of leptin in the AD/Lep/HF cohort (at least in females) may identify the factors that protect against metabolic syndrome even in the presence of a thrifty hepatic phenotype.

## Conclusions

In this study, we have established that both prenatal undernutrition and postnatal leptin treatment lead to a thrifty liver programme, and explain this as a nonspecific response to growth restriction from any cause. We further show that this thrifty programming is associated with increased expression of inflammatory markers and also with a downregulation of antigen presentation genes that may thus lead to immunosuppression. We propose a new model for developmental programming of obesity involving competing thriftiness and set point programmes, which resolves several conflicts between the data from male and female rats, and may apply more generally to other models of obesogenic programming.

## Methods

### Liver samples

The experimental design utilised in this study has been described previously [17-19]. Briefly, virgin Wistar rats were time-mated and assigned to two nutritional groups: ad-libitum (AD) or undernourished (UN, 30% of ad-libitum). After birth, female AD and UN pups were randomized to receive either saline (Sal) or recombinant rat leptin (Lep, 2.5 μg/g · d) on neonatal days 3-13. After weaning (day 22), saline-or leptin-treated AD and UN offspring were weight matched and placed on either standard rat chow or a high fat (HF) chow (Research Diets No. 12451; 45% energy as fat) for the remainder of the study. At postnatal day 170 rats were fasted overnight and killed by halothane anaesthesia followed by decapitation. This results in a balanced factorial 2×2×2 design with 8 treatment groups, n = 8 animals per group. Liver samples were immediately removed (collected from the same lobe) and immediately snap frozen in liquid nitrogen and stored at−80°C for later analysis. Time of culling was rotated within and between groups to avoid circadian confounders, and was restricted to a narrow window between 9-11 am, at the start of the light phase of the standard 12/12 dark/light cycle.

### RNA extraction

RNA from whole liver tissue was prepared using Trizol reagent (Sigma) according to the manufacturer’s protocol. Briefly, for each individual, 25 mg of frozen liver tissue was homogenised in 1 ml Trizol, followed by chloroform extraction. The aqueous supernatant containing the RNA was retained and the RNA precipitated with isopropanol. RNA pellets were washed with 50% ethanol, air dried and resuspended in RNAse-free water (Milli-Q). RNA concentration and integrity was assayed with a Nanodrop 1000 spectrophotometer and an Agilent 2100 bioanalyser.

### Microarray hybridisation and scanning

Array profiling was performed using the RatRef-12 oligonucleotide platform (Illumina). This comprises 21,910 probes covering the vast majority of the rat transcriptome. Probe labelling, hybridisation, washing and scanning were performed according to the manufacturer’s protocols using the Illumina Total Prep kit (Applied Biosystems). Briefly, first strand cDNA was synthesised in a total volume of 20 μl with the supplied reagents. The complete first strand product was used for second strand synthesis, followed by column purification. The purified product was then used for *in vitro* transcription using T7 polymerase. Biotin-16-dUTP was incorporated during this step, resulting in a biotinylated cRNA (complementary RNA) probe suitable for hybridisation. Probe integrity was verified using the Nanodrop 100 and Agilent 2100, as for the initial RNA samples. Labelled cRNA (1.5 μg) was hybridised to the array overnight at 55°C in a total volume of 30 μl of the manufacturer’s hybridisation buffer, followed by post-hybridisation stringency washing and scanning (BeadArray Reader, Illumina) using the manufacturer’s standard protocols.

### Data extraction and QC

Array data was extracted and presence/absence calls performed using BeadStudio (Illumina), and subsequently normalised in Lumi using a variance-stabilising transformation. For each set of eight measurements for each gene/experimental condition combination, the single measurement furthest from the mean was excluded. This has the effect of robustly eliminating noisy outlier measurements while maintaining a fully balanced experimental design. After outlier exclusion, an initial filter was applied to remove non-expressed genes (defined as those called as absent in > =75% of the data set). This reduced the initial set of 21,910 probes to 8,497 liver-expressed genes for further analysis.

### ANOVA analysis, gene classification and filtering

One-way ANOVA analysis and gene list filtering was performed using Kensington Discovery Edition (Inforsense). Benjamini/Hochberg correction was used to control for multiple testing, with a 5% false discovery rate (FDR). This yielded a list of 2221 probes showing significant regulation in at least one experimental cohort. Following Zhou et al. 2011 [38], these genes were then classified via a three-way ANOVA looking at all three experimental factors (A = maternal diet, B = leptin treatment, C = postweaning diet) and all four potential interaction terms (AB/BC/AC/ABC interactions). Three-way ANOVA was performed using GNU Octave.

First, genes with a significant three-way interaction after FDR correction were assigned to group ABC. Next, genes with no significant three-way interaction, but where one or more of the two-way interaction terms was significant after FDR correction were assigned to groups AB/AC/BC as appropriate. Finally, genes with significant effects for each individual experimental factor and no confounding interaction term were assigned to groups A/B/C as appropriate. The categories are not necessarily exclusive: for example a gene showing additive non-interacting effects of both maternal diet and leptin treatment is included in both categories A and B. However, a gene with a significant interaction term, i.e. where both factors are significant but the effects do not combine linearly, would instead fall into category AB. This iterative method of classification ensures that a gene is only included in a given category if there are no confounding higher-order interaction terms.

### DAVID analysis

DAVID analysis was performed using the web interface available at http://david.abcc.ncifcrf.gov/summary.jsp using default parameters for clustering stringency and the Illumina rat chipset (RatRef-12_V1_0_R4_11222119_A) as the reference list from which to calculate functional cluster enrichment.

### Phenotypic measurements

Plasma leptin was measured by in-house radioimmunoassay as described previously (Vickers et al., Endocrinology 2001). Fasting plasma insulin, C-peptide and total ghrelin were measured using commercially available kits (rat insulin ELISA, Cat# 10-1124-10; Mercodia, Uppsala, Sweden; C-peptide RIA, cat# RCP-21 K; Linco Research Inc., St. Charles, MO; Ghrelin RIA, cat# GHRT-89HK, Linco Research). Fasting plasma glucose was measured using a YSI glucose analyzer (Model 2300; Yellow Springs Instrument Co., OH, USA). Plasma FFAs, glycerol and triglycerides were measured in-house using standard colorimetric assays.

### Availability of supporting data

The expression array data set supporting the results of this article is available via LabOnline, doi:10.6070/H4X63JVV https://mynotebook.labarchives.com/share/Leptin/MjMuNHwyOTA2NC8xOC03L1RyZWVOb2RlLzMzMzM5NTUwNjh8NTkuNA==. Further supplementary data files are provided online as follows:

## Competing interests

The authors declare that they have no competing interests.

## Authors’ contributions

PDG, MHV and NAA conceived the study. PDG and MHV provided the tissue samples used for array analysis and carried out the measurements of phenotypic parameters. TJM performed sample extraction and microarray hybridisation. PJIE, TJM and BMS carried out the microarray analysis and functional clustering. SG and CAS advised on the functional interpretation of array results. PJIE drafted the manuscript. PJIE, BMS, NAA, MHV and TJM reviewed/edited the manuscript and all authors read and approved the final manuscript.

## Supplementary Material

Additional file 1: Table S1 Phenotypic parameters for the experimental series and 3-factor ANOVA analysis of significance. Click here for file

Additional file 2: Table S2 All genes found to show a significant expression change, classified by which factor(s)/and interaction(s) were significant. Individual tabs are given for each factor or combination of factors. Click here for file

Additional file 3: Table S3 Front tab: Cluster analysis of the 949 genes with a significant interaction between maternal diet and leptin. Further tabs show the annotation groups detected by DAVID as significantly enriched in each cluster. Click here for file

Additional file 4: Table S4 Weight data for both male and female experimental series at all time points from birth to sacrifice. Click here for file

Additional file 5: Table S5 Front tab: An expanded version of Table 3 (disease-associated genes regulated by early life programming: bold indicates “precursor genes” with altered expression in Un/Sal/Chow), with additional columns relating to known disease associations documented in OMIM and other databases. Remaining tabs show the annotation groups detected by DAVID as significantly enriched among disease-associated genes and precursor genes. Click here for file
